# Store-Operated Ca^2+^ Entry Contributes to Piezo1-Induced Ca^2+^ Increase in Human Endometrial Stem Cells

**DOI:** 10.3390/ijms23073763

**Published:** 2022-03-29

**Authors:** Vladislav Chubinskiy-Nadezhdin, Svetlana Semenova, Valeria Vasileva, Alla Shatrova, Natalia Pugovkina, Yuri Negulyaev

**Affiliations:** Institute of Cytology, Russian Academy of Sciences, Tikhoretsky Ave. 4, 194064 Saint-Petersburg, Russia; svsem@incras.ru (S.S.); vasileva.valeriia@gmail.com (V.V.); shatrova@mail.ru (A.S.); natalia.pugovkina@gmail.com (N.P.); yurineg@incras.ru (Y.N.)

**Keywords:** endometrial mesenchymal stem cells, cell migration, mechanosensitive channels, Piezo1 channels, store-operated Ca^2+^ channels, Ca^2+^ influx

## Abstract

Endometrial mesenchymal stem cells (eMSCs) are a specific class of stromal cells which have the capability to migrate, develop and differentiate into different types of cells such as adipocytes, osteocytes or chondrocytes. It is this unique plasticity that makes the eMSCs significant for cellular therapy and regenerative medicine. Stem cells choose their way of development by analyzing the extracellular and intracellular signals generated by a mechanical force from the microenvironment. Mechanosensitive channels are part of the cellular toolkit that feels the mechanical environment and can transduce mechanical stimuli to intracellular signaling pathways. Here, we identify previously recorded, mechanosensitive (MS), stretch-activated channels as Piezo1 proteins in the plasma membrane of eMSCs. Piezo1 activity triggered by the channel agonist Yoda1 elicits influx of Ca^2+^, a known modulator of cytoskeleton reorganization and cell motility. We found that store-operated Ca^2+^ entry (SOCE) formed by Ca^2+^-selective channel *ORAI1* and Ca^2+^ sensors *STIM1*/*STIM2* contributes to Piezo1-induced Ca^2+^ influx in eMSCs. Particularly, the Yoda1-induced increase in intracellular Ca^2+^ ([Ca^2+^]_i_) is partially abolished by 2-APB, a well-known inhibitor of SOCE. Flow cytometry analysis and wound healing assay showed that long-term activation of Piezo1 or SOCE does not have a cytotoxic effect on eMSCs but suppresses their migratory capacity and the rate of cell proliferation. We propose that the Piezo1 and SOCE are both important determinants in [Ca^2+^]_i_ regulation, which critically affects the migratory activity of eMSCs and, therefore, could influence the regenerative potential of these cells.

## 1. Introduction

Mesenchymal stem cells derived from a wide range of adult tissues, such as bone marrow, umbilical cord, peripheral blood and adipose tissue, are widely applied to treat various diseases. In the past decades, new sources of adult stem cells, including the placenta [1], fallopian tube [2], amniotic fluid [3,4], amniotic membrane [5] and endometrium [6,7,8] were established. Special interest was given to the stem cells isolated from female reproductive organs, particularly the endometrium [6,7,8]. The endometrium is a dynamic tissue involved in the synchronized functions of cellular proliferation, differentiation and menstrual shedding [9]. It constantly sheds its top layer of cells during menstruation, which is later regenerated completely by the basal layer of the endometrial tissue, suggesting the existence of a very high potential stem cell population [10]. Endometrial stem cells (eMSCs) are a heterogeneous cellular population composed mainly of stromal and epithelial cells of the endometrial gland that demonstrate an MSC-specific, fibroblast-like morphology [11]. It was found that eMSCs can be differentiated into various types of cells such as myocytes, cardiomyocytes, osteocytes, adipocytes and neurons [12]. Due to their high availability, these cells are considered as one of the reliable, non-invasive and very valuable substrates for therapeutic interventions. Therefore, profound knowledge of the molecular mechanisms that control the migration and differentiation of eMSCs is of clinical importance.

The mechanical action on main cellular functions, such as proliferation, migration, gene expression and differentiation, is very important for stem cell transplantation since stem cells are constantly exposed to different mechanical stimuli and forms of physical stimulation, including sound, light, temperature, mechanical force and even gravity [13,14,15,16]. At the same time, it is established that ion channels, particularly mechanosensitive channels, are directly or indirectly involved in the transduction of all forms of physical stimulation [17]. Piezo1 and Piezo2 molecules were identified as a novel family of mechanosensitive cation channels by Coste et al. [18]. Further, Piezo channels were shown to participate in a variety of mechano-dependent physiological processes, and they were found to play a crucial role in the transmission of physical stimulation to intracellular signaling pathways [19,20]. The signaling functions of Piezo are mainly mediated by their ability to conduct Ca^2+^ ions, which are known to be crucial, secondary messengers in living cells. There is substantial evidence connecting the handling of intracellular calcium concentration ([Ca^2+^]_i_) to the normal physiology and pathophysiology of endometrial stromal cells. It is established that different Ca^2+^-permeable ion channels are involved in decidualization and play a key role in embryonic functions [21,22,23,24].

The participation of Ca^2+^ channels, TRP channels, cAMP-induced cytosolic Ca^2+^ levels and Ca^2+^-binding proteins suggests that [Ca^2+^]_i_ is tightly connected with uterine function [21,25,26,27,28]. However, owing to technical and ethical problems, ion channel investigations have been restricted in human endometrial stem cells isolated from human tissue. Here, we use human eMSCs to address the role of mechanosensitive Piezo1 channels in [Ca^2+^]_i_ level regulation in endometrial cells. Given the significant role of the temporal and spatial Ca^2+^ regulation in a variety of main cellular functions, including cell motility, we also examined the role of Piezo1 in endometrial cell migration.

## 2. Results

### 2.1. Expression of Mechanosensitive Piezo1 Channels in Human Endometrial Mesenchymal Stem Cells (eMSCs)

**Figure 1:**
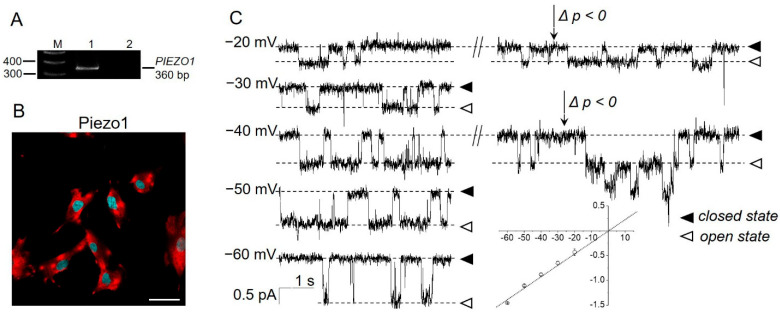
Presence of Piezo1 in eMSCs. (**A**) RT-PCR analysis revealed the presence of *PIEZO1* mRNA. M—size marker, Line 1—primers specific for *PIEZO1* amplified the PCR product of the expected size (360 bp), Line 2—RT-PCR negative control in which reverse transcriptase was omitted. Shown is cropped gel with enchanced contrast. (**B**) Immunofluorescent staining detected Piezo1 proteins (red channel) in eMSCs. No staining of the cells was observed after incubation of the cells with only fluorescent secondary antibodies (Supplementary Figure S2). Cell nuclei were counterstained with DAPI (blue channel). The scale bar is 50 µm. (**C**) The single-channel activity of Piezo1 induced by selective chemical Piezo1 agonist Yoda1 (10 µM in the pipette solution). Representative current recordings at different membrane potentials are shown. Holding membrane potentials are indicated near current traces, closed and open states indicate the baseline (“zero” current) and Piezo1 open state, respectively. Note that the application of “negative” pressure (*p* < 0, suction, indicated by the arrows) further increased the activity of the Yoda1-induced channels. The mean I–V relationship corresponded to single-channel conductance of 23.2 ± 1.3 pS (*n* = 6).

Piezo1 channels are found in various tissues and cell lines, including several human mesenchymal stem cells [29,30]. The mechanosensitive channel activity with biophysical properties close to the Piezo family was observed in the plasma membrane of eMSCs in response to membrane stretch (“negative” pressure application, suction [31]). Importantly, the Piezo channel family consists of two members [18], Piezo1 and Piezo2, and, of them, only Piezo1 can be activated by both “negative” or “positive” pressure application, whereas Piezo2 was recently reported to only be sensitive to low-threshold “positive” pressure [32]. Therefore, we hypothesized that Piezo1 proteins are likely molecular correlates of stretch-activated MS channels in eMSCs, and they could provide a physiologically relevant pathway for Ca^2+^ influx and, thereby, affect the [Ca^2+^]_i_ and Ca^2+^-dependent signaling processes. By consistently using PCR analysis and immunofluorescence, we first confirmed the expression of Piezo1 in eMSCs (Figure 1A,B).

To detect the functional activity of Piezo1 channels in the plasma membrane of eMSCs, we stimulated the channels in cell-attached patch-clamp experiments using a selective chemical activator of Piezo1 [33]: a small synthetic molecule Yoda1. No significant background channel activity was observed in the control patches (without Yoda1, *n* > 100, Supplementary Figure S1). Importantly, the presence of Yoda1 in the patch pipette activated the unitary inward currents (Figure 1C) with biophysical properties similar to those previously reported for stretch-activated currents in eMSCs [31]. Moreover, the application of “negative” pressure in the presence of Yoda1 further increased Yoda1-induced channel activity (Figure 1C, the effect is demonstrated on −20 and −40 mV holding potentials). Taken together, our data strongly support the hypothesis of Piezo1 channel expression and its activation by Yoda1 in human eMSCs.

### 2.2. Piezo1 Channels as Ca^2+^ Entry Pathway in eMSCs

**Figure 2:**
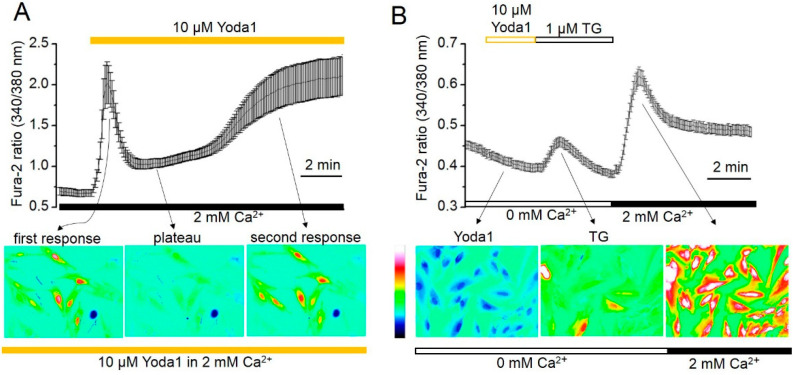
Yoda1 induces the two-component Ca^2+^ entry in human eMSCs. (**A**) Shown are typical Ca^2+^ responses from the representative experiment (*n* = 3) following the Yoda1 application to the cells. Measurements were performed in individual cells using the imaging system. [Ca^2+^]_i_ is plotted versus time. Each point is a mean ± SD from 19 cells. The pseudocolor ratiometric calcium images correspond to the level of [Ca^2+^]_i_ in the cells at different timepoints after addition of 10 µM Yoda1. (**B**) Yoda1 induces no Ca^2+^ increase in Ca^2+^-free solutions. Shown are typical Ca^2+^ responses from the representative experiment (*n* = 3) with Yoda1 addition followed by TG application. Each point is a mean ± SD from 22 cells. The pseudocolor ratiometric calcium images correspond to the level of [Ca^2+^]_i_ in the cells after the application of 1 µM TG and 2 mM Ca^2+^.

It is well known that the activation of mechanosensitive, cation-selective channels results in Ca^2+^ entry from the extracellular environment to the cytoplasm of the living cells. Thereby, mechanosensitive channels play a significant role in the regulation of [Ca^2+^]_i_. Here, we assume that stretch-activated MS channels identified as Piezo1 proteins promote Ca^2+^ influx and affect the [Ca^2+^]_i_ in eMSCs. To test this hypothesis, we loaded cells with Fura-2AM, and Ca^2+^ responses induced by selective chemical Piezo1 activator Yoda1 were estimated using an AxioObserver.Z1 inverted microscope imaging system (see Section 4). In our experiments, a large increase in [Ca^2+^]_i_ was observed after the addition of 10 µM Yoda1 to the extracellular solution. Interestingly, the Yoda1-induced Ca^2+^ response consisted of two distinct components: the first, a fast component of [Ca^2+^]_i_ growth developed immediately and quickly declined (plateau) after the addition of Yoda1 to the external solution. Then, several minutes later, a second increase in [Ca^2+^]_i_ (delayed component), which lasted the length of the [Ca^2+^]_i_ registration, was observed (Figure 2A). A calcium ionophore, ionomycin (at a final concentration of 10 μM), was used as a positive control for the reaction (Supplementary Figure S4). Particularly, the addition of ionomycin further increased [Ca^2+^]_i_, indicating that the second Yoda1-induced component was not caused by disruption of the cell membrane integrity followed by passive calcium entry. Wash-out of extracellular Ca^2+^ with a Ca^2+^-free solution abrogated the delayed Yoda1-induced increase in [Ca^2+^]_i_, indicating the extracellular nature of the second calcium entry. In addition, as the experiments lasted up to 30 min, we specifically checked the potential damage of the cells by UV light [34]. No visible morphological changes in the cells exposed to UV radiation were observed (Supplementary Figure S3).

Figure 2 indicates that the kinetics of Yoda1-induced Ca^2+^ responses resemble the classical Ca^2+^ responses observed due to Ca^2+^ release from internal Ca^2+^ storage with subsequent store-operated Ca^2+^ entrance (SOCE) via the plasma membrane (see, for example, [35]). Therefore, firstly, we examined the potential Yoda1 participation in Ca^2+^ release from Ca^2+^ stores. Figure 2B demonstrates no [Ca^2+^]_i_ increase after Yoda1 application to cells in calcium-free solution, suggesting no Ca^2+^ release from intracellular Ca^2+^ stores in response to Piezo1 activation. Notably, the further addition of thapsigargin (TG), a specific, irreversible inhibitor of endoplasmic reticulum (ER) Ca^2+^-ATPases in the same ion conditions resulted in TG-induced Ca^2+^ release, as was previously documented elsewhere [35]. Figure 2B shows the typical Ca^2+^ increase induced by TG in a Ca^2+^-free solution and the following store-operated Ca^2+^ entry, indicating the existence of functional Ca^2+^ store release and the following activation of SOCE in eMSCs.

### 2.3. Expression of Orai1, STIM1 and STIM2 in eMSC Cells

**Figure 3:**
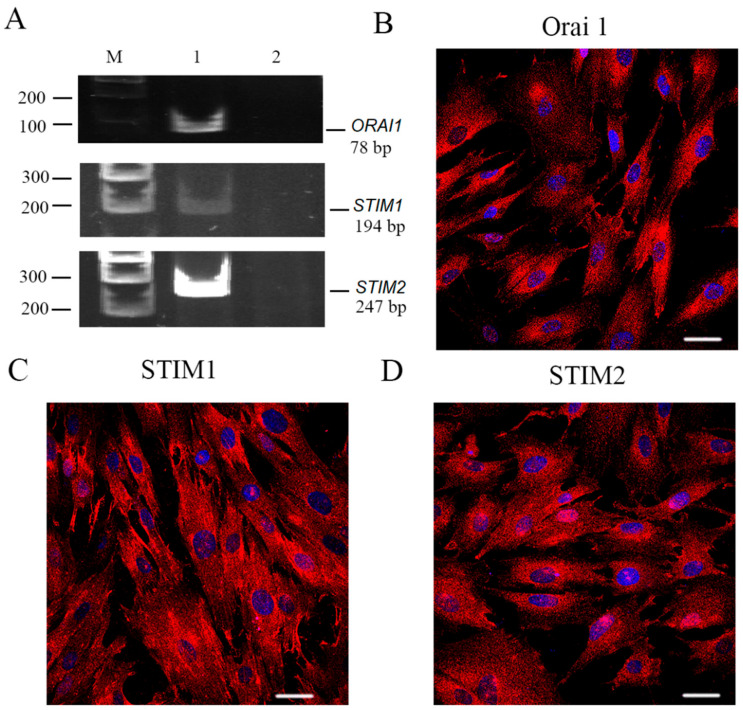
*ORAI1*, *STIM1* and *STIM2* expression in human eMSCs. (**A**) RT-PCR analysis of *ORAI*, *STIM1* and *STIM2* expression in eMSCs. M—size marker. Line 1—primers specific for *ORAI1*, *STIM1* and *STIM2* amplified the PCR products of the expected sizes (78, 124 and 247 bp, correspondingly). Line 2—RT-PCR negative control in which reverse transcriptase was omitted. Shown are cropped gels with enhanced contrast. Original gel is shown in Supplementary Figure S5. The distribution of *ORAI1* (**B**), *STIM1* (**C**) and *STIM2* (**D**). Immunoreactivity in eMSCs was examined with confocal microscopy. No staining of the cells was observed after incubation of the cells with only fluorescent secondary antibodies (Supplementary Figure S2). Scale bar is 50 µm.

SOCE is a general Ca^2+^ influx via the plasma membrane which is triggered by G-protein-coupled receptors (GPCRs) activation and the following IP3-mediated depletion of endoplasmic reticulum Ca^2+^ stores. SOCE represents a complex of proteins that includes calcium-selective channel *ORAI* and the endoplasmic reticulum-associated Ca^2+^ sensor STIM. It is known that the *ORAI* family has three homologs, *ORAI1*, *2* and *3*, and *ORAI3* is exclusively present in mammals [36]. The *STIM1* protein has another homolog: *STIM2*. Therefore, using PCR analysis, we investigated the expression of the *ORAI1–3* and *STIM1* and *2* in endometrial stem cells. PCR primers were designed as indicated in Material and Methods. RT-PCR analysis revealed the 78 bp products, 194 bp products and 247 bp products that correspond to *ORAI1*, *STIM1* and *STIM2* transcripts, respectively, which are expressed in eMSCs (Figure 3A), whereas the presence of *ORAI2* and *3* mRNA was not detected (Supplementary Figure S5).

To evaluate the *ORAI1*, *STIM1* and *STIM2* protein localization and spatial distribution in cells, the specific antibodies and immunofluorescence confocal microscopy were utilized. As presented in Figure 3, the immunofluorescence signal was detected in both cytoplasm and plasma membrane of the cells marked with the antibodies recognizing *ORAI1* (Figure 3B), *STIM1* (Figure 3C) and *STIM2* (Figure 3D).

### 2.4. Contribution of SOCE in Piezo1-Induced Ca^2+^ Influx

**Figure 4:**
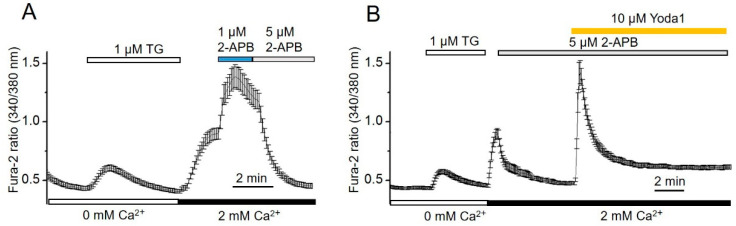
2-APB suppresses the store-operated Ca^2+^ entry and abrogates the second (delayed) phase of Yoda1-induced Ca^2+^ increase in eMSCs. (**A**) Shown are typical Ca^2+^ responses from the representative experiment (*n* = 4) following the 1 μM and 5 μM 2-APB application on eMSCs. Measurements were performed in individual cells with the imaging system. [Ca^2+^]_i_ is plotted versus time. Each point is a mean ± SD from 17 cells. (**B**) 2-APB suppresses both SOCE and the delayed phase of Piezo-mediated Ca^2+^ entry in eMSCs. Each point is a mean ± SD from 13 cells.

Preliminary experiments showed that the calcium responses to selective chemical stimulation of mechanosensitive Piezo1 channels kinetically resemble that of store-operated Ca^2+^ entry activated by TG (Figure 2). Therefore, we hypothesized that SOCE may contribute to the increase in [Ca^2+^]_i_ induced by Piezo1 channel activation. To verify this hypothesis, a pharmacological SOCE inhibitor, 2-aminoethyl diphenylborinate (2-APB), was used. At the beginning of the experiment, Ca^2+^ was removed from the external cell medium, which immediately led to a reduction in the level of [Ca^2+^]_i_. Subsequent application of TG caused a rapid [Ca^2+^]_i_ growth followed by a rapid decline (Figure 4A). The addition of 2 mM Ca^2+^ to the external solution led to typical growth of [Ca^2+^]_i_ as a result of SOCE activation. It is especially noteworthy that the application of 1 µM 2-APB did not suppress the SOCE-activated Ca^2+^ influx, whereas 5 µM 2-APB completely abolished it (Figure 4A).

Therefore, to eliminate the potential contribution of SOCE in Piezo-induced Ca^2+^ influx, 2-APB was used in the concentration of 5 µM. Figure 4B demonstrates that the addition of Yoda1, together with 2-APB, to the extracellular solution does not prevent the occurrence of the first component of [Ca^2+^]_i_ increase but suppresses the second (delayed) one, suggesting the SOCE contribution in Piezo1-mediated Ca^2+^ increase.

### 2.5. Role of SOCE and Piezo1 in eMSC Migration and Proliferation

It is well known that MSCs possess a homing capacity, can move into damaged areas and help in tissue regeneration [37,38,39,40,41]; therefore, MSCs represent a significant tool for regenerative medicine [42,43,44,45]. Lately, there were several reports that proper control of Ca^2+^ signaling is important for effective cell migration. However, how Ca^2+^ coordinates the necessary factors for effective cell moving, key signaling pathways and signaling molecules is still elusive. Here, we investigate the likelihood of participation of Piezo1- or SOCE-induced Ca^2+^ currents in eMSC migration.

**Figure 5:**
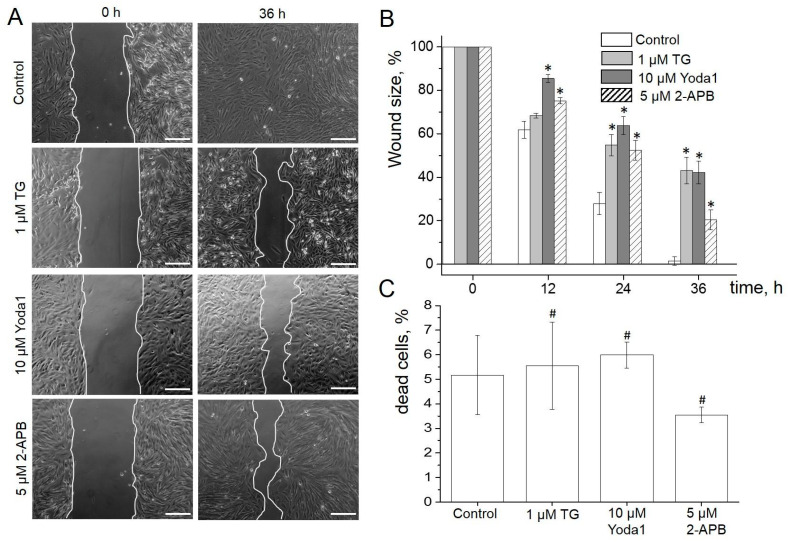
SOCE and Piezo1 in eMSCs migration. (**A**) Shown are representative images of the experimental wounds at the beginning (0 h) and the end of the wound healing assay (36 h). The scale bar is 200 µm (**B**). The eMSC wound healing dynamics in the presence of TG, Yoda1 or 2-APB in the culture media. Wound sizes are shown as mean ± SEM (*n* = 3–4 for each condition). * significantly different compared to control, Student’s *t*-test with Welch’s corrections, *p* < 0.05. (**C**) No significant changes in the viability of eMSCs were observed. Shown is the mean percent (±SD, *n* = 3) of dead cells for each experimental condition. #—not significantly different compared to control, two-sample Student’s *t*-test (*p* < 0.05).

The wound healing assay, together with time-lapse imaging, was used to address this challenge. To avoid some possible, uncontrolled effects caused by cell damage, the wound in the eMSC culture was created by the removal of a silicone insert, as described in Materials and Methods. Figure 5A presents typical images showing the wound areas at the starting time point and 36 h after eMSC treatment with 1 µM of TG and 10 µM Yoda1 (see also Supplementary Video S1 showing the time-lapse process of eMSC wound healing). Wound healing area analysis revealed that both SOCE and Piezo1 activation significantly inhibits the wound healing processes. Particularly, the control wounds were fully closed 36 h after the beginning of the experiments, whereas about 40% of the wounds in the presence of Yoda1 or TG remained uncovered by the cells (Figure 5B, Supplementary Table S4). Interestingly, the cell cultivation in the presence of 5 µM 2-APB also resulted in the suppression of the wound healing process (Figure 5B, Supplementary Table S4). To exclude the potential toxic effect of the compounds, we determined the eMSC viability using PI staining at the end of the cell migration experiments. It was established that the percentage of viable eMSCs in the population did not significantly change even after 48 h of treatment with TG, Yoda1 or 2-APB compared to control cells (Figure 5C, Supplementary Table S4). Thus, the data demonstrated that the observed effect of the suppression of wound healing processes is not caused by the cytotoxic effects of the reagents.

**Figure 6:**
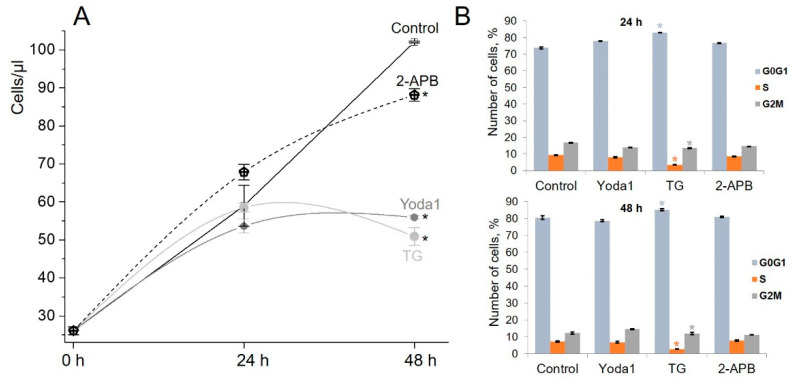
Impact of Yoda, TG and 2-APB treatment on eMSC proliferation and cell cycle distribution. (**A**) Growth curves of eMSCs in the presence of 5 µM 2-APB, 10 µM Yoda1 or 1 µM of TG. The data are presented as mean ± SD (*n* = 3), * significantly different compared to control (Student’s *t*-test, *p* < 0.05). (**B**) The percentage of cells in the G0/G1, S and G2/M phases. The data are presented as mean ± SD (*n* = 3), * significantly different compared to control (Student’s *t*-test, *p* < 0.05).

Wound healing is a standard behavior of epithelial and endothelial cells, as well as mesenchymal cells, that is firstly activated to increase their motility in order to settle the injured area and then, together with their ability to differentiate and to secrete various biologically active factors and molecules [46], they also demonstrate a high, proliferative rise to ensure the successful completion of the wound repair process [47]. Our data revealed the suppression of the wound healing induced by Yoda1, TG and 2-APB; therefore, we assumed that these reagents potentially affect the rate of cell proliferation, and this may partially underlie the observed decrease in cell motility. Our experiments showed that all reagents suppressed the growth of eMSCs, and the pronounced result was observed after 48 h of cell treatment (Figure 6A). The cell cycle FACS analysis showed the significant eMSCs accumulation in the G0/G1 phase after TG treatment (Figure 6B, see, also, Supplementary Table S5). At this time, the pattern of phase distribution of the cells treated with Yoda1 or 2-APB showed no accumulation of the cells in the particular phase of cell cycle.

## 3. Discussion

It is well known that all eukaryotic cells are mechanosensitive since they are subjected to various environmental mechanical forces, such as gravity, tension, compression and shear. These mechanical forces impact various cellular functions down to the fate definition of cells. The mechanosensitive Piezo channels are capable of sensing mechanical force and transform force and pass signals into cells in the form of Ca^2+^ currents on a millisecond time scale. It appears that these evidently specific force transducers exist throughout the numerous cells. Although Piezo channels discovery happened relatively recently, extensive evidence has already been found for their significance in many aspects of cellular life.

Here, we report that the mechanically gated ion channel Piezo1 is expressed in human endometrial stem cells. We also show that the small-molecule modulator of Piezo1 channels, Yoda1, induces large Ca^2+^ entry in eMSCs. Importantly, the Yoda1-induced Ca^2+^ entry has two distinct components that are separated in time. The kinetics of Yoda1-induced Ca^2+^ response are similar to those induced by TG in numerous cells, particularly: (i) TG-mediated [Ca^2+^]_i_ growth caused by store depletion followed by (ii) store-operated Ca^2+^ entry. However, our experiments demonstrated that the application of Yoda1 in a Ca^2+^-free solution has no effect on [Ca^2+^]_i_, whereas the following addition of TG results in the increase of [Ca^2+^]_i._

Both TG-activated store depletion and store-operated Ca^2+^ entry were detected in Ca^2+^ measurements carried out on eMSCs. We also identified that store-operated Ca^2+^ entry in eMSCs could be formed by *ORAI1*/STIM1 or *ORAI1*/*STIM2* proteins. In our experiments, a specific blocker of SOCE, 2-APB, suppressed the TG-induced SOCE. Moreover, 2-APB also blocked the second (delayed) component of Yoda1-induced Ca^2+^ increase, suggesting the SOCE is participating in mechanosensitive Ca^2+^ signaling in cells. These findings correlate with the data which show that store-operated Ca^2+^ entry can participate in Ca^2+^ influx induced by the other agonists, for example, ATP [30,48].

Aside from this, some scientific reports recently indicated that Ca^2+^ entry mediated by mechanosensitive channels is regulated by a diversity of major proteins which are located in the cellular membrane or endoplasmic reticulum. It was shown that mechanosensitive channels can be physically connected with some Ca^2+^ regulatory proteins [49]. For example, Piezo and SERCA were discovered to cross-talk to each other through physical interaction in endothelial cells. SERCA2 was demonstrated to suppress Piezo1-induced HUVEC migration via a 14-residue linker region. The linker mutations disrupt the cooperation between Piezo1 and SERCA2, and, consequently, the mechanical activation of Piezo1 channels is essentially reduced [50]. The main function of sarco/endoplasmic reticulum Ca^2+^-ATPase (SERCA) is to transport Ca^2+^ from the cytosol into the sarco/endoplasmic reticulum. SERCA inhibition induces the store-operated Ca^2+^ influx. One could, therefore, assume that Piezo1, by closely interacting with SERCA2, can activate SOCE that contributes to the Piezo1-induced Ca^2+^ entry.

Our results show that TG decreases the migration of eMSCs. These data are consistent with those presented by the other authors that show that TG decreases the proliferation, migration and invasion of human adenocortical carcinoma SW-13, NCI-H295R [51] and esophageal carcinoma EC109 and TE12 cell lines [52]. TG also suppressed ACC xenograft tumor progression in vivo [51]. TG is considered as a potential antineoplastic drug; however, it is also highly toxic to normal cells [53]. Interestingly, we did not observe any effect of TG on eMSC viability even after 48 h of incubation (Figure 5C, Supplementary Table S4).

In addition, we demonstrated that selective chemical activation of Piezo1 by Yoda1 decreases the migration potential of eMSCs. In our previous study, we showed that Yoda1 dose-dependently inhibits migration of transformed mouse fibroblasts [54] that was accompanied by F-actin assembly and stress fiber formation. It is noteworthy that the effect of selective Piezo1 activation by Yoda1 on cellular motility remains rather controversial. Particularly, the increase of migration of mesenchymal stem cells in response to Yoda1 was reported, and the effect was mediated via the release of ATP and the activation of P2 receptor signaling [30]. In contrast, the decrease of migration of keratinocytes in response to Yoda1 was observed, and the specific mechanism which is the localized increase of the retraction of the rear end of the cells induced by Yoda1 was proposed [55]. Another mechanism of Piezo-controlled cell motility was reported in *Dictyostelium* where Piezo channels were shown to control whether cells migrate with blebs or pseudopods [56]. The genetic depletion of Piezo1 expression can also have opposite effects on cellular motility: that is, the reduction of cell migration [57,58,59] or the increase of migratory properties of the cells after Piezo1 knockdown [60,61]. The variability of the effect of the manipulation of Piezo1 activity on cell migration is more likely explained by the coupling of Piezo1 with different intracellular signaling pathways in the cells.

Somatic stem cells permanently regulate their self-renewal and lineage commitment by combining different environmental signals to support tissue homeostasis. Although multiple chemical and biological signals that control stem cell behavior were recovered, whether stem cells can directly respond to mechanical stimuli remains unclear. This investigation has made some significant findings: first, Piezo1 was identified in eMSCs, and it acts as a molecular mechanism that contributes to mechano-induced Ca^2+^ entry in eMSCs. Second, *ORAI1*/*STIM1*/*STIM2* proteins are expressed and mediate store-operated Ca^2+^ influx in eMSCs and act as a downstream mechanism that participates in Yoda1-induced Ca^2+^ entry. Third, both Piezo1-induced and store-operated Ca^2+^ entry suppress the eMSC migration, the rate of cell proliferation and induce cell cycle arrest without any effects on cell viability. We believe that the knowledge about the mechanisms underlying Piezo1-induced Ca^2+^ signaling and their role in eMSC migratory activity will help to develop novel strategies aimed at modulation of the regenerative potential of eMSC for tissue repairing.

## 4. Materials and Methods

### 4.1. Cell Cultures and Reagents

The endometrial mesenchymal stem cell (eMSC) line isolated from the desquamated endometrium of the menstrual blood of a healthy donor (line no. 2804, [11]) was a gift from the Department of Intracellular Signalling and Transport, Institute of Cytology, RAS. The cells had properties that are typical for MSCs, including fibroblast-like morphology, multipotency and the expression of standard surface markers [62]. Cells were grown in DMEM/F12 cultural medium (Gibco, Waltham, MA, USA) with 10% fetal bovine serum (HyClone, Logan, UT, USA) and 1% penicillin/streptomycin (Gibco, Waltham, MA, USA). The cells were maintained in culture flasks at 37 °C in 5% CO_2_. 2-[(2,6-dichlorophenyl) methylsulfanyl]-5-pyrazin-2-yl-1,3,4-thiadiazole (Yoda1) was purchased from Tocris (Tocris, Abington, United Kingdom, cat. no. 5586). 2-aminoethyl diphenylborinate (2-APB) and thapsigargin (TG) were purchased from Sigma-Aldrich (Sigma-Aldrich, St.Louis, MO, USA, cat. no. D9754 and T9033, respectively)**.**

### 4.2. Total RNA Extraction and Reverse Transcriptase (RT)-PCR

Total RNA was isolated using the RNeasy Mini Kit (Qiagen, Germantown, MD, USA). The PCR primers were designed using the GeneRunner v5.0.59 software. The primer sequences are represented in Table 1. To avoid false positive results due to genomic contamination of the samples, the primers spanned an intron at the genomic level. PCR was performed in the volume of 10 µL using 1 µL diluted (1:3) cDNA, 0.3 µM of each primer, 200 µM dNTPs, 2 mM MgCl_2_, 1 unit Hot-Taq polymerase and 1 × Hot-Taq polymerase buffer (Sileks, Moscow, Russia). In negative control experiments, MMLV reverse transcriptase was omitted. PCR reaction products were subjected to electrophoresis on a 6% polyacrylamide gel and visualized by UV fluorescence after ethidium bromide staining.

### 4.3. Immunofluorescence

For immunofluorescent staining, the cells, preliminary plated on glass coverslips, 2–3 days before the experiments, were fixed with 3.7% paraformaldehyde in phosphate-buffered saline (1 × PBS, 10 min) and permeabilized with 0.25% Tween-20 (10 min at room temperature, RT). Non-specific binding of the antibodies (Abs) was blocked by incubation with 10% goat serum for 1 h at RT. Then cells were incubated with goat anti-rabbit polyclonal Abs against Piezo1 (Novus Bio, Centennial, CO, USA, cat. no. NBP1-78537) or rabbit polyclonal Abs against *ORAI1* (Alomone Labs, Jerusalem, Israel, cat. no. ACC-060) or Abs against *STIM1* (Alomone Labs, Jerusalem, Israel, cat. no. ACC-063) or *STIM2* (Alomone Labs, Jerusalem, Israel, cat. no. ACC-064) at 4 °C overnight. Goat anti-rabbit (GAR)-Cy3 Abs (Santa Cruz, Dallas, TX, USA) were used for secondary detection. Cell nuclei were counterstained with DAPI (Sigma-Aldrich, St.Louis, MO, USA, final concentration was 0.1 µg/mL) and then the samples were mounted using Vectashield mounting medium (Vector Lab. Inc., Burlingame, CA, USA). As a control, staining of the cells only with fluorescent secondary Abs (GAR-Cy3, Santa Cruz, Dallas, TX, USA) was used. Images were obtained using the Olympus FV3000 laser scanning confocal inverted microscope (Olympus Corporation, Shinjuku, Tokyo, Japan) with 40×/1.3 NA oil immersion objective and a specific combination of laser/detectors.

### 4.4. Electrophysiology

Single currents were recorded using cell-attached configuration, as described previously [63]. Briefly, the patch-clamp set-up was based on the Axopatch 200B operational patch-clamp amplifier (Molecular Devices, San Jose, CA, USA) and Axon Digidata 1550A (Molecular Devices, San Jose, CA, USA) analog–digital converter controlled by a Windows-based personal computer with installed Axon PClamp 10.7 Software Suite (Molecular Devices LLC, San Jose, CA, USA) for data acquisition, processing and analysis. Patch pipettes were pulled from borosilicate glass capillaries (BF-150-86-10, Sutter Instruments, Novato, CA, USA) to a resistance of 10–15 MOhm when filled with the extracellular solution containing (in mM): 145 NaCl, 2 CaCl_2_, 1 MgCl_2_ and 10 HEPES/TrisOH. Potassium bath solution containing 145 KCl, 2 CaCl_2_, 1 MgCl_2_ and 10 HEPES/TrisOH was used to nullify the resting membrane potential of the cells. The pH of all solutions was maintained at 7.3. All experiments were performed at RT. The single-channel recordings were processed and analyzed in Clampfit software (part of Axon PClamp 10.7 Software Suite). Single-channel conductance values were defined by the slope of the current–voltage relationship (I–V) after linear approximation. Averaged conductance values are given as the mean ± SEM (*n*—number of experiments).

### 4.5. Ca^2+^ Imaging

eMSCs were seeded on cover slides 2–3 days before Ca^2+^ measurements. On the day of the experiment, cells were washed with a serum-free medium and loaded for 35 min with a 4 μM Fura-2AM probe (Thermo Fisher Scientific, Waltham, MA, USA) in the dark at RT. Then, cells were washed, and the cover slides were transferred into a perfusion chamber. Cell imaging was obtained using an AxioObserverZ1 inverted microscope (Carl Zeiss MicroImaging GmbH, Oberkohen, Germany) with a Carl Zeiss Fluar 40×/1.3 Ph3 NA oil objective. Fura-2AM fluorescence was excited every 5 s sequentially by the light of 340 nm or 380 nm from an illuminator with a Lambda DG-4 high-speed wavelength switcher (Sutter Instrument Co., Novato, CA, USA). Filter set 21 HE (Carl Zeiss MicroImaging GmbH, Oberkohen, Germany) was used (excitation BP 340/30, emission BP 510/90). “Physiology” plugin of AxioVision 4.8.2 software (Carl Zeiss MicroImaging GmbH, Oberkohen, Germany) was used for data acquisition and analysis. Calcium imaging recordings were subjected to the subtraction of the background fluorescence. Fura-2AM 340/380 nm ratio values are given as mean ± SD (*n*—number of the cells).

### 4.6. Quantification of Cell Migration (Wound Healing Assay)

For the wound healing assay, trypsinized eMSCs were seeded into Ibidi Culture-Inserts 2 Well (Ibidi GmbH, Gräfelfing, Germany, total number of cells was 70,000 per insert) pre-installed in 24-well culture plates to form the experimental wounds (gaps of the standardized sizes). The cells were cultured for a further 24 h to allow cell spreading, then, the inserts were removed, and the plates were transferred to the stage of a Carl Zeiss AxioObserver Z1 (Carl Zeiss Microimaging GmbH, Oberkohen, Germany) microscope equipped with the set-up for long-term live-cell imaging under stable culture conditions (controlled humidity, 37 °C and 5% CO_2_). The images of the experimental wounds were acquired automatically (every 2 h; total time of the experiment was 48 h) using the “Multidimensional Acquisition” plugin of Carl Zeiss Axiovision 4.8.2 Software (Carl Zeiss Microimaging GmbH, Oberkohen, Germany). The sizes of experimental wounds were calculated manually using the “Measure Area” tool in ImageJ software (NIH, Bethesda, MD, USA), and the wound sizes were normalized to the size of the initial wound (at 0 h starting measurement). Wound sizes are shown as mean ± SEM (*n*—number of independent experiments). Data were compared using a two-sample Student’s *t*-test (for data with unequal variances, Welch’s correction was introduced); *p* < 0.05 was considered as significant.

### 4.7. FACS Analysis

Detached with trypsin/EDTA solution, eMSCs were suspended in growth medium and utilized for viability and cell cycle analysis as described previously [64]. Briefly, 0.05 mg/mL of propidium iodide (PI) was added to the eMSCs and exposed to flow cytometry (FACS) analysis. The cells gated as PI-negative were used for growth curve generation. For cell cycle analysis, saponin (0.2 mg/mL), RNAse (0.25 mg/mL) and PI (0.05 mg/mL) were applied to cell suspension, mixed gently and incubated for 1 h in the dark at RT. No less than 3000 events were gathered for viability assay and 15,000 events for cell cycle analysis. Cytometric analysis was conducted with CytoFLEX S flow cytometer (Beckman Coulter, Brea, CA, USA) equipped with Cytexpert software (version 2.0). Data were compared using a two-sample Student’s *t*-test (*p* < 0.05 was considered as significant) and are presented as mean ± SD.

### 4.8. Statistics

Statistical analysis was performed using GraphPad Prism 6.0 software (GraphPad Software, San Diego, CA, USA). All data were tested for normal distribution using Shapiro–Wilk’s test and homogeneity of variances (Levene’s test). The particular, statistical criteria used for means comparison are described in the relevant sections of Materials and Methods; *p* < 0.05 was considered as significant.

## Figures and Tables

**Table 1:** Primers for RT-PCR analysis.

Gene	Forward Primer	Reverse Primer
*hPIEZO1*	3′-CCAGAACAGGTATCGGAAG-5′	5′-TGCTGTACCAGTACCTGCTG-3′
*hORAI1*	5′-GACCTCGGCTCTGCTCTC-3′	5′-GGTGGGTACGTGGTCAG-3′
*hORAI2*	5′-GCGGAAGCTCTACCTGAG-3′	5′-CCACATTGGGCAGGATGC-3′
*hORAI3*	5′-CACGTCTGCCTTGCTCTC-3′	5′-ATGTTGCTCACAGCTTCAATG-3′
*hSTIM1*	5′-GGCAGTCCGTAACATCCAC-3′	5′-TTGTATAC TTCTGATGACTTCC-3′
*hSTIM2*	5′-ATGGTGGAATTGAAGTAGAGG-3′	5′-TTCCTTTGACATTGTTGTCTC-3′

## Data Availability

The data presented in this study are available within the article, in Supplementary Information and upon request.

## Supplementary Materials

The following supporting information can be downloaded at: https://www.mdpi.com/article/10.3390/ijms23073763/s1.
