# Supplementary material for: Store-Operated Ca2+ Entry Contributes to Piezo1-Induced Ca2+ Increase in Human Endometrial Stem Cells
Source: Int J Mol Sci. 2022 Mar 29;23(7):3763. doi: 10.3390/ijms23073763 (PMC8998223; doi:10.3390/ijms23073763)
Supplement: Supplementary file 1 [file ijms-23-03763-s001.zip › Supplementary data.pdf]

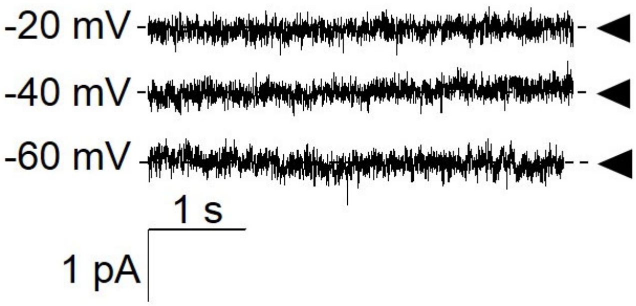

Supplementary Figure S1. Representative cell-attached current recordings (from n>100) demonstrating no background channel activity in the absence of Yoda1 in the pipette solution. Holding membrane potentials are indicated near traces, baseline indicates zero current level.

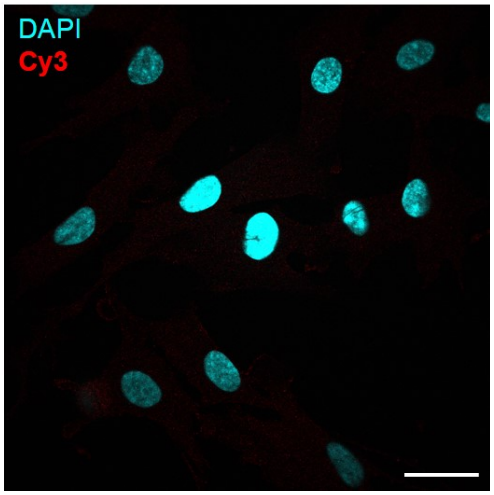

Supplementary Figure S2. No fluorescent staining of the cells is observed when cells were incubated only with secondary antibodies (1:200, GAR-Cy3, 1h at room temperature). Cell nuclei were counterstained with DAPI. The scale bar is 30 μM.

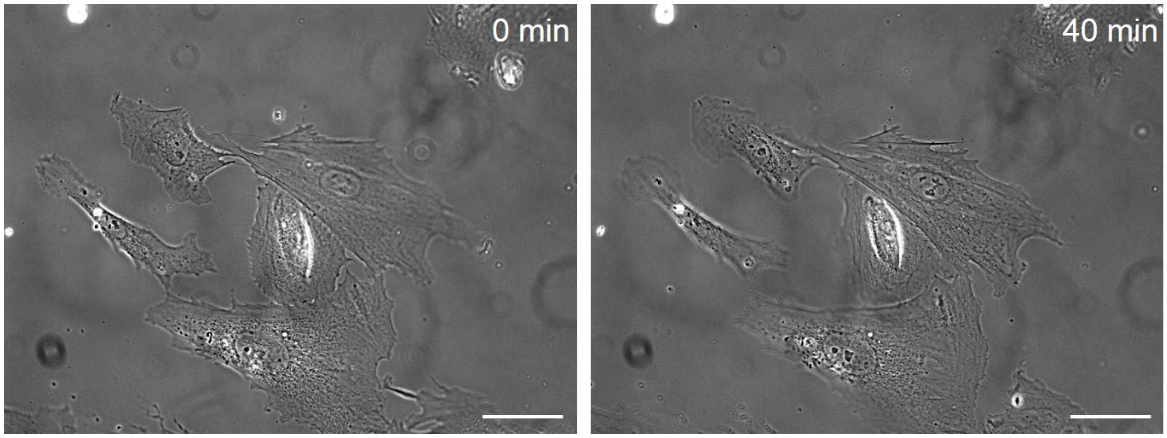

Supplementary Figure S3. Brightfield images of eMSCs demonstrating cell morphology before (0 min) after 40 min of $[Ca^{2+}]_i$ imaging using UV light of 340/380 nm length. Imaging conditions are identical to those used for calcium measurements. Particularly, the cells were exposed every 5 sec to 400 ms of 340 nm UV and then 400 ms of 380 nm UV light. The scale bar is 50 μm.

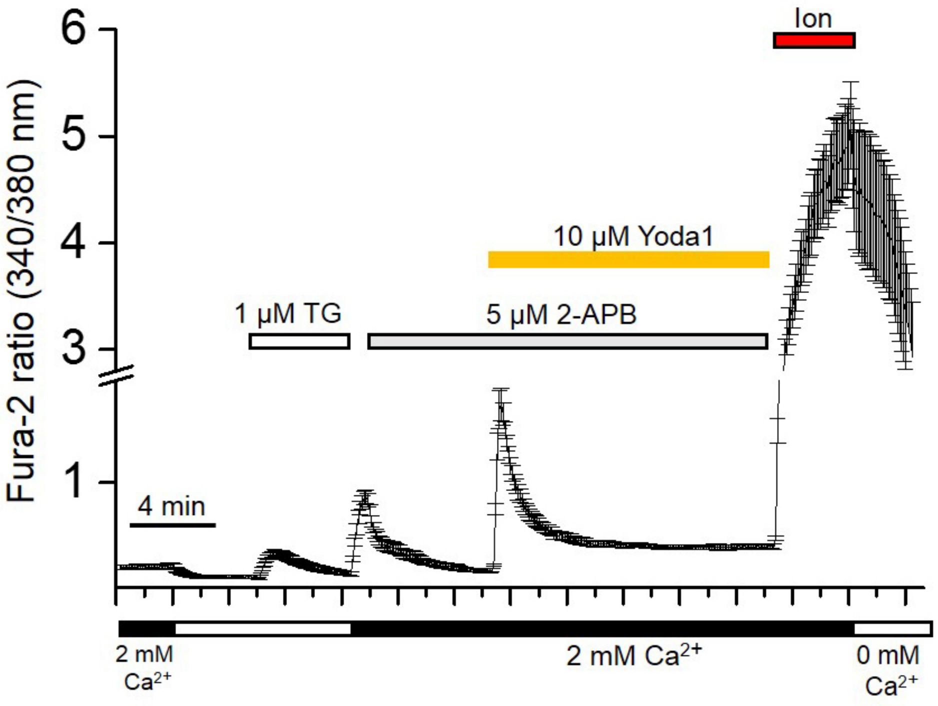

Supplementary Figure S4. Ionomycin (Ion, 10 μM) further increased Ca2+ entry in cells after addition of Yoda1. Wash-out with Ca2+-free solution resulted in-interruption of Ionomycin-induced Ca2+ entry in eMSCs. Shown are typical Ca2+ responses from the representative experiment (n=4). [Ca2+]i is plotted versus time. Each point is a mean ± S.E. from 13 cells.

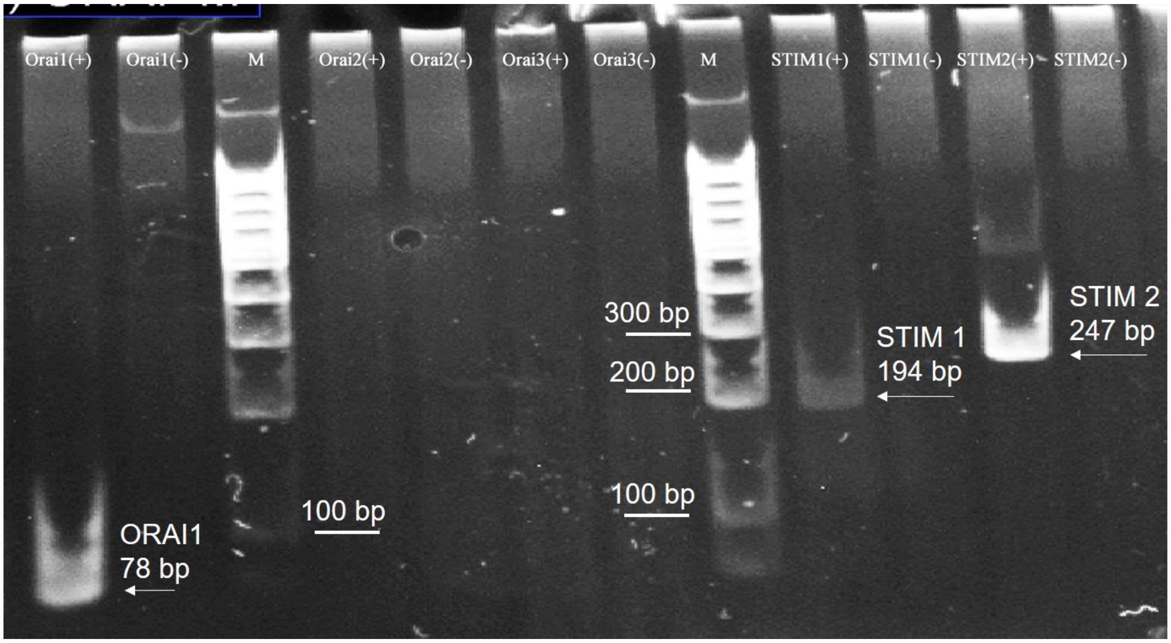

Supplementary Figure S5. Original gel demonstrating the presence of ORAI1, STIM1 and STIM2 and the absence of ORAI2 and ORAI3 transcripts in eMSCs.

	The remaining wound sizes, in %. mean ± S .E. M.			% of dead (PI-positive) cells, mean ± S.D.
**Timepoints:**	**12 h**	**24 h**	**36 h**	**48 h**
Control	61.77 ± 3.96	27.86±5.08	1.53 ± 1.93	5.17 ± 1.61
1 μM TG	68.35 ± 1.08	57.80 ± 4.92	43.11 ± 6.08	5.55 ± 1.78
10 μM Yoda1	85.43 ± 1.87	63.76 ± 4.09	42.26 ± 5.26	5.99 ± 0.54
5 μM 2-APB	75.18 ± 1.35	52.50 ± 4.61	20.36 ± 4.51	3.55 ± 0.33

Supplementary Table S1. Wound sizes from representative wound healing assay at 12, 24 and 36 h time points and the number of dead cells after 48 h incubation with the studied compounds.

**24 h**				
	Control	10 μM Yoda1	1 μM TG	5 μM 2-APB
GO/G1	73.82 ± 0.47	77.99 ± 0.20	83.01±0.21	76.80 ± 0.25
S	9.35 ± 0.24	8.13 ± 0.39	3.44 ± 0.09	8.51± 0.21
G2M	16.81 ± 0.22	13.87± 0.18	13.53± 0.31	14.67± 0.03
**48 h**				
GO/G1	80.42 ± 1.02	78.63± 0.47	85.13±0.64	80.87± 0.48
S	7.27± 0.30	6.84± 0.62	2.83± 0.01	7.86± 0.36
G2M	12.29 ± 12.29	14.52± 0.15	12.02±.64	11.26 ± 0.11

Supplementary Table S2. The phase distribution of the eMSCs treated with Yoda1, TG or 2-APB after 24 and 48 h. Data are presented as Mean % ± S.D. (n=3).

Supplementary Video 1. Representative movies of eMSCs wound healing process in control and in the presence of 1 μM TG, 10 μM Yoda1 or 5 μM 2-APB in the media. The total length of the experiment is 48 h, the time between frames is 2 h.
